# Exploration of the optimal modularity in assembly line design

**DOI:** 10.1038/s41598-022-24972-2

**Published:** 2022-11-27

**Authors:** Vladimir Modrak, Zuzana Soltysova

**Affiliations:** Faculty of Manufacturing Technologies, Technical University of Kosice, 080 01 Pres̆ov, Slovakia

**Keywords:** Mechanical engineering, Computational science

## Abstract

It is widely accepted that a proper structural modularity degree of assembly processes in terms of mass customization has a positive effect on their efficiency because it, among other things, increases manufacturing flexibility and productivity. On the other hand, most practical approaches to identify such a degree is rather based on intuition or analytical reasoning than on scientific foundations. However, the first way can be used for simple assembly tasks, but in more complex assembly processes, this method lags behind the second. The purpose was to create a methodology for selection of optimal modular assembly model from among a predefined set of alternatives. The methodology is based on exploration of the relations between modularity measures and complexity issues as well as the relationship between structural modularity and symmetry. Especially, the linkage between modularity and complexity properties has been explored in order to show how modularization can affect distribution of the total structural complexity across the entire assembly line. To solve this selection problem, three different methods are preliminary suggested and compared via a series of numerical tests. The two of them present the novel contribution of this work, while the third method developed earlier for the purpose of finding and evaluating community structure in networks was adapted for a given application domain. Based on obtained results, one of these method is prioritized over another, since it offers more promising results and precision too.

## Introduction

In today’s mass customization assembly systems, most companies use modular patterns in design processes as they offer more flexibility to react to market demands than non-modular ones and have a positive effect on their productivity^1,2^. Beyond that, next generation of manufacturing factories which is all about intelligent connected supply chains often include modular and flexible manufacturing systems. One of the other reasons why modular design structures are favoured over integrated ones lies on the assertion that modular designs are a useful means of managing complexity^3^. Modularly organized networks are characterized, apart from other properties, by functional segregation, integration and hierarchical nature^4^. Assembly line networks that are of interest in this paper belong to this network type.

In general, architecture of assembly lines and their modular or integral character depends on topological designs of products. An *assembly line structure* (ALS) consists of a number of different work stations purposed to put together all the parts and subassemblies of specified products. Each of the stations may combine several production inputs—components to produce an output. In modular process configurations, assembly tasks are in principle assigned to intermediate sub-assemblers instead to doing all those tasks by the final assembler. Such functional segregation enables to offer higher variety of products by different combinations of assembly stations (modules) than integral architecture of ALSs. On the other hand, a modular assembly process design brings disadvantages related to the dispersion of activities and resources^5^. Therefore, designers should make a trade-off between modular and integral architectures of ALSs^6^. Naturally, to find the right compromise between modular and integral design it requires being able to apply the concept of optimal network modularity. Otherwise, it may lead to error-prone decisions. In order to introduce this crucial concept of this work it is useful to say at the outset that the notion of optimal modularity is rarely used in engineering design^7^. The modularity optimization problem has quite long history, and it includes several methods for detecting communities in complex networks in order to estimate the optimal number of communities for a given network. For this purpose, Newman and Girvan^8^ proposed the *Optimal Modularity measure* (Q), which is defined by the fraction of the edges in the network that connect between nodes within a module minus the expected value of the same quantity in a network with the same assignment of nodes into modules but random connections between the nodes. According to Guimerà and Amaral^9^ an ideal partition of a network into modules must comprise many within-module edges and as few as possible between-module edges. These important features of the optimal modularity concept evoke also a necessity to differ between the two similar properties of the networks that are *optimal network modularity* and *network granularity level*.

The second notion usually refers the 'grain size' i.e., the size and the detail of the system elements before or after their decomposition^10^ in a sense that “a higher granularity is characterized by an increase of interlinked functions in the model^11^”. In this context, system elements can be analyzed at different decomposition levels or they are partitioned at the same level, respectively. The second situation corresponds to the considered case in this work for expressing granularity level of ALSs. Accordingly, the granularity level of assembly line expresses the extent to which the process structure can be decomposed into modules which are arranged in parallel, and/or in series^12^. In line with this, ALS which consists of only one un-partitioned assembly station is taken here as fully integral archetype of the network. By contraries, modular ALS is composed of cohesive internally nested assembly stations that interact with one another of the same network. Taking in account the definition of process modularity provided by Blecker and Abdelkafi^13^, such type of modularity is comprehended as “dividing a large process into smaller sub-processes that can be designed and then operated independently while still ensuring that the whole process fulfils its objectives”. When comparing the semantics of this definition with the core meaning of the term *network granularity level*, it can be easily seen that the content of both terms is identical. Moreover, their content differs considerably from the notion of *optimal network modularity*. Therefore, the network granularity is considered here as *relative process modularity*, and its quantitative measures will be used as the complementary tools to arrange alternative ALSs from the lowest level of granularity to the highest.

The *optimal network modularity* concept is from the viewpoint of exploration and exploitation at least frequently associated with many types of networked systems (see, e.g.^14–16^), as well as it is related to product architecture design^7,17,18^. Therefore, there are different views to the concept, and a pertinent question is what we regard as optimal architectural design in a given domain of interest. To provide a clear and unambiguous statement to it is rather precarious as the concept of system design modularity is still in its evolutionary phase with a lack of consistent definitions related to the notion of optimal modular design^19^.

A broader goal of this paper is to find a suitable tool for selection of optimal modular ALS model from among a predefined set of identified alternatives. To solve this kind of selection problem, it is proposed to use a newly developed approach for identification of the optimal modularity. In order to compare this approach against a possible concurrent method, the *optimal modularity* measure Q has been adapted for a given purpose.

The main objective of this research is investigation of the optimal modularity in designing ALSs with focusing on the following two research questions (RQs):

RQ 1: To what extent the existing optimal modularity index Q developed for non-technical systems is applicable for the purpose of identification of optimal process structure modularity?
RQ 2: What benefits the proposed optimal modularity indicator Om_1_ can bring for the specific technical domain?

The reminder of this paper is organized as follows. The following “Related work” section presents a brief overview of the related research in this domain. Thereafter, a theoretical framework for investigation is introduced focusing on the relationship between modularity measures and complexity issues as well as the relationship between modularity and symmetry in the context of ALSs. After that, the three potential optimal modularity measures are described with examples of their applications illustrated on a simple ALS. Moreover, the same section provides a validity assessment of the three indicators. Then, common features and general findings related to two of the three optimal modularity concepts are extracted. Subsequently, an applicability of the proposed approach is demonstrated through a real case. Finally, crucial additional issues are discussed and future research directions are outlined.

## Related works

### Relationship between process modularity and product modularity

There is no doubt that modular design has many advantages against integral one. Therefore, modularity as a design principle helps designers to find appropriate degree of product or process modularity. In addition, modularity is also considered as a powerful organizing principle of the evolutionary processes of both artificial and natural complex systems^20^. According to Ulrich^21^, products or processes can be modular or integral depending on whether or not, and how, their functions are allocated to the modules. Some researchers claim that right degree of process modularity may improve its efficiency, increase manufacturing flexibility and reduce production costs^22–24^. Kusiak^25^ points that process modularity is tightly connected with product modularity, and outlines a framework for integral product and process design. Yelles-Chaouche et al^26^ pointed out in a context of optimization of reconfigurable manufacturing systems that an important research task lies in optimizing the number of process modules which will able to fulfil the variety needs. Parker^27^ identifies three different modularity facets which are functional biding, decomposability, and interface standardization. The first of them is directly related to the research presented here. The main idea of functional binding is one-to-one mapping of functions to sub-processes in order to reduce their interdependence. This process or product modularity aspect closely corresponds to the systems design methodology known as Axiomatic Design introduced by Suh^28^. Vickeri at al.^29^ investigated relationships between product modularity, process modularity, and new product introduction performance. Results of their research showed that alignment of product and process modularity has a positive effect on new product introduction performance. Several authors (see, e.g., works of^30,31^) argue that product modularity increases production process efficiency, flexibility and agility. On the other hand, process modularity accelerates new product introduction and helps raise quality of products^32,33^.

### Approaches to measurement of process modularity

In the context of process modularity measurement, it seems important to take into account especially the fact that existing related measurement concepts can hardly be applied across disciplines, even though they appear self-evident within a single discipline^34^. On the other hand, many of such concepts can be very helpful in developing similar methods. Among them, Frenken and Metriczky^35^ proposed a construct of optimal level of modularity, which minimizes the time required to globally optimize a system. Abdelkafi^19^ transformed so called Module Independence indicator, developed by Blackenfelt^36^, into Cross-Module Independence index in order to measure process modularity. AlGeddawy and ElMaraghy^37^, proposed a granularity index to quantify the quality of Design Structure Matrix, which is applicable in wider design domains. This indicator is calculated for all granularity levels of a hierarchical clustering obtained through cladistics analysis^38^. Rough set theory presents other related research approach, by which Yao^39^ defines information-theoretic granularity metric. This granularity measure is defined based on the Shannon entropy to quantify partitions generated under the same network. Other related works^40,41^ are focusing on process modularity measures and provide additional context for the relationships between process modularity and complexity, as well as insight into the nexus of process modularity and product modularity. All of the mentioned approaches are more or less inteded to quantify the relative modularity of manufacturing processes and systems.

### Optimal modularity as a design objective

Nevertheless, the most important problem regarding the modularity of process structures is their optimal modularity level. As it is known, optimal modularity does not equate to maximal relative granularity^42–44^. In this context Alkan et al.^45^ investigated trade-off between complexity and optimal modularity in modular assembly supply chain networks. Efatmaneshnik and Ryan^7^ proved that optimal modularity can be achieved through a balanced modularization by using a concept of structural symmetry in the distribution of the module sizes. The same authors outlined, that modularization remains more of an art than a science if optimal modularity measure is not applied in this effort. Shoval et al.^46^ proposed to identify optimal modularity of assembly supply chains arranged in serial lines. Their approach is based on the assumption that for any given sequence of the assembly operations the expected cost is reduced when the assembly operations are divided as evenly as possible among the subassemblies. The mentioned method identifies optimal structure of subassemblies by applying cost-based criterion for determining optimal assembly supply chain from alternative ones. This complementary approach differs from the proposed novel method in this way that it is based on the stable number of process operations, while the proposed approach determines the number of structures based on the stable number of input components and different number of process operations. According to Mehrsai et al.^47^, extension of modularity into operations and resources allows to create alternative structures of supply networks by splitting their performances into modules and adjusting them as required. In this context, Sharmelly and Ray^48^ state that “modularity saves us lot of time and cost because if the designs are modular then you don’t need to design the whole system all over again if anything goes wrong.”

## Theoretical framework for investigation

As mentioned in section 1, theoretical framework for investigation of the modularity issues in this study is based on the relationship between modularity measures and complexity issues as well as the relationship between modularity and symmetry. Especially, two mutuaally related types of complexity measures, *overall design complexity* and *module level complexity* are used as a basis through which the proposed concept of optimal modularity is contextualized. Finally, in order to show that symmetric process structures are favoured among modular ALSs, so called Symmetric index is introduced in this section. In addition, a methodical determination of alternative ALSs based on the number of initial input components entering into the assembly line is introduced from the beginning. The purpose of this is to obtain alternative ALSs with varying configuration patterns.

The following notations will be used throughout this paper:

i: Is the number of input components,
int: Is a specific module,
n: Is the number of modules,
R: Is the sum of the relations within a module,
T: Is the sum of all relations in a given network,
deg(v)_i_: Is the degree of vertex of (v)i in graph G, while G consists of a set of V vertices {V} = {v_1_, v_2_, …, v_v_},
N_j_: Is the number of couplings per column, j = 1, …, K, where K is the number of columns in design matrix,
α: Is multi-tier complexity coefficient,
β: Is manufacturing network nodes coefficient,
γ: Is manufacturing network links coefficient,
T_i_: Is i-th tier level,
N_s_: Is s-th node,
LK_ij_: Is j-th network link in i-th tier level,
O_m_: Is the number of orbits of graph G under the action of automorphism group on the set of vertices {V} = {v_1_, v_2_, …, v_v_}, which are partitioned into k equivalent orbits {O_1_, . . ., O_k_} containing just one node,
Aut(G): Denotes the set of automorphisms of G,
L: Is the number of all edges in the network,
ls: Is the number of internal edges in module s,
ws_out_: Is the number of output edges in module s,
ws_in_: Is the number of input edges in module s.

**Figure 1:**
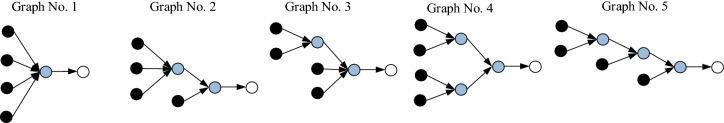
All possible alternative assembly line structures with four input components.

1. *Formalized specification of ALSs* Modular assembly line is defined as a set of workstations interconnected by a material handling system consisting of transport and stock devices^49^. It is assumed that order of assembly operations cannot be changed, i.e., pre-determined precedence constraints are respected. Alternative assembly line structures are modelled for given purpose as multifurcuting rooted directed convergent trees (see Fig. 1), in which the leaf nodes present input components entering the assembly operations (internal nodes), while the root represents completed product. As can be seen from Fig. 1, the assembly station of Graph No. 1 can be decomposed into limited number of modules/stations arranged in parallel and series. The specific properties of such graphs are formulated as follows:
  (i) Each internal node has at least two children.
  (ii) All possible alternative assembly line structures are assumed those with identical number of input components. The numbers of all possible ALSs when a number of input components is specified can be exactly determined through the integer sequence A000669^50^.
  (iii) All possible ALSs with the same number of input components create a class of ALSs (class#i), where *i* stands for the number of input components.
  (iv) Each class of ALSs with i ≥ 3 contains exactly one *graph of 'star' type* (see Graph No. 1 in Fig. 1). Moreover, each the class consists only of one *graph of 'comb' type* with maximum depth of graph (see Graph No. 5 in Fig. 1), while depth of the graph determines the maximum distance of the leaves from the root. It can be also noted that internal nodes of the comb graphs have necessarily at least one leaf child.
  (v) Each class of ALSs with even number of input components has at least one fully symmetric graph. Fully symmetric graph is defined here as a tree in which the subtrees formed by descendants of child nodes of the same parent are identical^51^. In other words, a tree graph is fully symmetric from head to tail if its pendant subtrees are isomorphic in the sense that they have the same morphological characteristics, but it is assumed here that even though the star graph is symmetric, it is not considered as fully symmetric. Accordingly, ALSs of class#4 consists of one fully symmetric structure (Graph No. 4) with the two levels of internal nodes.
  (vi) Each process module of assebly line structure encompasses one or more assembly operations, and such a module is represented only by a single internal node.
2. *Relative modularity measures of ALSs* As mentioned in previous section, only a few metrics to quantify relative modularity of ALSs are available in literature^40,41,52,53^. It is prioritized to select for given purpose two of them, namely *cross-module independence* (CMI) indicator and *relative network modularity* (RNM) measure, since the both are directly applicable for ALSs. These estimators can be described as follows:
  (i) CMI is defined through the ratio of the sum of relations inside all modules to the sum of all relations, and may be formally expressed as follows^36^:
    1 $$CMI = 1 - \mathop \sum \limits_{int = 1}^{n} \frac{{R_{int} }}{T},$$
    where int denotes a specific module, n stands for the number of modules, R is the sum of the relations within a module, and T is the sum of all relations in a given ALS model. Modules of ALSs are represented in the trees by internal nodes.
  (ii) RNM measure is expressed through the following formula^41^:
    2 $$RNM = \frac{n}{{\mathop \sum \nolimits_{i = 1}^{v} deg\left( v \right)_{i} \cdot log_{2} deg\left( v \right)_{i} }},$$
    where *deg(v)*_*i*_ is the degree of vertex of (v)_i_ in graph *G*, while *G* consists of a set of *V* vertices {V} = {v_1_, v_2_,…,v_v_}, and *n* is the number of modules in the network.
    When applying these two formulas, e.g., on Graph No. 3, then relative modularity values are obtained:
    $$CMI = 1 - \frac{5}{6} = 0.167,$$
    $$RNM = \frac{2}{{1 \cdot log_{2} 1 + 1 \cdot log_{2} 1 + 1 \cdot log_{2} 1 + 1 \cdot log_{2} 1 + 1 \cdot log_{2} 1 + 3 \cdot log_{2} 3 + 4 \cdot log_{2} 4}} = 0.157$$
3. *Overall design complexity measures of ALSs* A key objective during an initial design phase is to build an entire conceptual model of ALS that minimizes overall system complexity^54^. In general, there is expected that better modularity aims at increasing the efficiency by reducing complexity^52,55,56^. It is fair, taking into account also the assertion that the higher relative modularity increases the overall complexity^57^. This relation works also for the alternative ALSs, as shown further on in this paragraph.

For this purpose, we firstly adapted so called *architectural design complexity* (ADC) measure^56^ which is based on Boltzmann's entropy conception and Axiomatic Design theory. Following Axiomatic Design theory, the construct of this measure makes an assumption that distribution of functional couplings in the system’s structure gives a good opportunity to reflect design complexity. The functional couplings are in the given case identified between design parameters (DPs) and process variables (PVs). DPs and PVs are both rather conceptual categories used in various ways in different application domains. Design parameters, in terms of assembly of mass customized production, are assumed to be elements of a final design solution including input components that satisfies the specified functional requirements of individual customers. Process variables in initial design phase are comprehended as structural properties of process models created by mapping the design parameters.

ADC is defined using formula^56^:

3 $$ADC = \sum (Nj\cdot\ln Nj),$$

where *Nj* is the number of couplings per column, *j* = *1,…, K*, *K* is the number of columns in design matrix.

Moreover, *manufacturing flow complexity* (MFC) proposed by Crippa et al^58^ was used to assess overall design complexity. This alternative measure of overall complexity of ALSs assumes a positive, linear relationship between tiers, nodes, links and complexity, and it is expressed as follows:

4 $$MFC = \alpha \cdot \mathop \sum \limits_{i = 1}^{n} T_{i} + \beta \cdot \mathop \sum \limits_{s = 1}^{m} N_{s} + \gamma \cdot \mathop \sum \limits_{i = 1}^{n} \mathop \sum \limits_{j = 1}^{k} LK_{ij} ,$$

where α is multi-tier complexity coefficient (α ≥ 0), β is manufacturing network nodes coefficient (β ≥ 0), γ is manufacturing network links coefficient (γ ≥ 0), T_i_ is i-th tier level, N_s_ is s-th node, LK_ij_ is j-th network link in i-th tier level. If no weight is assigned to this coefficients, then it is assumed that α = β = γ = 1.

An example of application of these two formulas is provided in the next Section.

In order to demonstrate that the higher relative modularity of ALSs increases their overall complexity, the both relative modularity measures along with ADC and MFC measures are numerically compared using ALSs of class#4 from Fig. 1 and ALSs of class#5 from Appendix 1. Obtained values are numerically compared in Table 1.

**Table 1:** Comparison of the relative modularity and overall complexity values.

	CMI	RNM	ADC	MFC
**i = 4**				
No.1	0	0.086	13.6	14
No.2	0.167	0.157	22.1	17
No.3	0.167	0.157	20.2	17
No.4	0.29	0.21	27.1	19
No.5	0.29	0.21	**31.3**	**20**
**i = 5**				
No.1	0	0.065	18.8	16
No.2	0.143	0.12	29.9	19
No.4	0.143	0.12	25.81	19
No.3	0.143	0.125	27.7	19
No.7	0.25	0.17	41.61	22
No.9	0.25	0.17	37.2	22
No.5	0.25	0.17	34.92	21
No.6	0.25	0.17	39.68	22
No.8	0.25	0.17	33	21
No.10	0.333	0.21	49.88	24
No.11	0.333	0.21	44.69	24
No.12	0.333	0.21	**54.13**	**25**

Significant values are in [bold].

Table 1 clearly shows the expected relation between the relative modularity and overall complexity of the alternative ALSs.

(4) *Module level complexity measures of ALSs* In order to indicate topological properties of ALSs adequately, i.e. by capturing their inherent relations between the complexity and structural modularity without losing sight of the specifics related to given graphical models, it is further assumed that “the overall complexity is lower than or equal to the sum of all individual complexities^59^”. Then, so called *module level complexity* (MLC) measures can be derived from formulas (3) and (4) in simplified way as follows:
  5 $$MLC_{1} = \frac{{\sum (Nj\cdot{\text{ ln}}Nj)}}{n},$$
  6 $$MLC_{2} = \frac{{\alpha \cdot \mathop \sum \nolimits_{i = 1}^{n} T_{i} + \beta \cdot \mathop \sum \nolimits_{s = 1}^{m} N_{s} + \gamma \cdot \mathop \sum \nolimits_{i = 1}^{n} \mathop \sum \nolimits_{j = 1}^{k} LK_{ij} }}{n}.$$

where *n* is the number of modules in the network.

This measures which assign individual theoretical complexities to internal nodes doesn’t reflect their real complexities, but bring complementary view to understand the essence of complexity in this domain. It has been also found out that if relative modularity of ALSs increases, then so called *module level complexity* of ALSs decreases. This auxiliary finding is presented in the next section.

(5) *Symmetry as a basis for morphological analysis of ALSs* Firstly, it is worth mentioning that modularity and symmetry are typical system attributes observed in almost every engineering and biological structure^60^. Moreover, their relation is often explored in one direction, questioning how the modularity is affected by the symmetry, but not the other way around. In this intention it was found that there exists a significant impact of graph symmetry on optimal modularity^61,62^. Based on this finding related to different types of graphs it is assumed that optimal modularity level of ALSs is positively affected by the symmetry. When one wants to measure symmetry in graphs, the best way how to obtain the highest measurement quality is to use *Symmetry index* S(G) developed by Mowshowitz and Dehmer^63^. This measure which is based on information content of a graph is specified as follows:
  7 $$S\left( G \right) = \frac{1}{{\text{v}}}\left( {\mathop \sum \limits_{m = 1}^{k} \left| {O_{m} } \right| \cdot log\left| {O_{m} } \right|} \right) + log\left| {Aut\left( G \right)} \right|$$

where O_m_ (1 ≤ m ≤ k) is the number of orbits of graph G under the action of automorphism group on the set of vertices {V} = {v_1_, v_2_,…,v_v_}, which are partitioned into k equivalent orbits {O_1_, . . . ,O_k_} containing just one node, and Aut(G) denotes the set of automorphisms of G.

**Figure 2:**
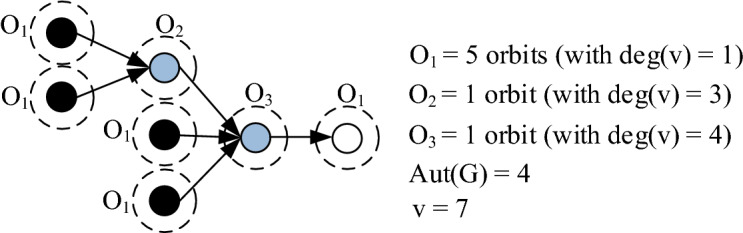
Parametrization of the selected graph from the viewpoint of symmetry quantification using S(G).

By applying this index using graph from Fig. 2, its symmetry value equels 3.66.

## Description and testing of optimal modularity measures

This section describes a proposed novel approach for measuring optimal modularity of ALSs as well as an existing technique widely accepted in the field of community networks which is adapted for the particular application. Contemporaneous testing of those three types of indicators, where the two of them (Om_1_ and Om_2_) differ in principle from Qd, makes it possible to capture the substance of their complementary nature, and to consider their potential suitability for the intended purpose.

### Proposition of optimal modularity measures

The aim of proposed methods is to find optimal partition of ALS networks into modules to minimize structural complexity of ALS, while the relative modularity is sufficiently high. By applying the *module level complexity* measures along with the relative modularity measures on alternative ALS models of class#4 (see Table 2 for i=4) it is evident that the structure No. 4 satisfies both the criteria in the best possible way. When comparing the values of MLC_1_ and MLC_2_ (from Table 2 for i=5) one can see that ALS No. 8 is identified as the best alternative according to MLC_1_, but based on MLC_2_, the most suitable alternatives are ALSs No. 10 and 11.

**Table 2:** An example of mutual relation between CMI, RNM, MLC_1_ and MLC_2_ indicators for class#4 and class#5.

	**CMI**	**RNM**	**MLC**_**1**_	**MLC**_**2**_
**i = 4**				
No.1	0	0.086	13.6	14
No.2	0.17	0.157	11.05	8.5
No.3	0.17	0.157	10.1	8.5
No.4	0.29	0.21	**9.03**	**6.3**
No.5	0.29	0.21	10.43	6.7
**i = 5**				
No.1	0	0.065	18.8	16
No.2	0.143	0.12	14.95	9.5
No.4	0.143	0.12	12.9	9.5
No.3	0.143	0.125	13.85	9.5
No.7	0.25	0.17	13.87	7.3
No.9	0.25	0.17	12.41	7.3
No.5	0.25	0.17	11.64	7
No.6	0.25	0.17	13.23	7.3
No.8	0.25	0.17	**11**	7
No.10	0.333	0.21	12.47	**6**
No.11	0.333	0.21	11.17	**6**
No.12	0.333	0.21	13.53	6.25

Significant values are in [bold].

In spite of the fact, that MLC_1_ and MLC_2_ (starting with ALS model of class#5 and higher) identify different optimal ALS models from the sets of alternative structures, both the measures satisfy the above mentioned criteria, while MLC_2_ seems to be less sensitive than MLC_1_.

In addition, it can be stated that structural relative modularity and module level complexity of ALSs are mutually related with a significant negative correlation. This finding is in line with the literature^64,65^ suggesting that proper modularization positively affects redistribution the total complexity across the system. Then, it is logical to assume that both the module level complexity measures could be used to determine optimal modularity measures of ALSs in direct way as inverse functions of MLC_1_ and MLC_2_ through the following formulas:

8 $$Om_{1} = \frac{n}{{\sum (Nj\cdot{\text{ ln}}Nj)}},$$

9 $$Om_{2} = \frac{n}{{\alpha \cdot \mathop \sum \nolimits_{i = 1}^{n} T_{i} + \beta \cdot \mathop \sum \nolimits_{s = 1}^{m} N_{s} + \gamma \cdot \mathop \sum \nolimits_{i = 1}^{n} \mathop \sum \nolimits_{j = 1}^{k} LK_{ij} }}.$$

In other words, the proposed *Optimal modularity* (Om_1_ and Om_2_) indicators encapsulate the fact that higher modularity level results in lover level of distributed complexity of ALSs.

**Figure 3:**
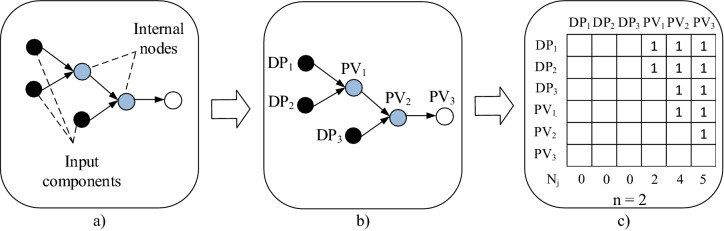
(**a**) ALS model; (**b**) ALS model described using AD notation; (**c**) related visibility matrix.

The following is an example of how these two indicators are calculated for the arbitrary ALS shown in Fig. 3a.

To enumerate Om_1_, ALS model from Fig. 3a is at first transformed into labelled graph in accordance to the Axiomatic Design notation as shown in Fig. 3b. Then, this graph is transformed into visibility matrix (see Fig. 3c) which denotes the dependencies of all possible path sequences. Subsequently, Nj values are determined (N_1_=N_2_=N_3_=0, N_4_=2, N_5_=4 and N_6_=5), and optimal modularity values Om_1_ and Om_2_ are obtained as shown below:

$$Om_{1} = \frac{2}{2 \cdot ln2 + 4 \cdot ln4 + 5 \cdot ln5} = 0.13$$

$$Om_{2} = \frac{2}{1 \cdot 3 + 1 \cdot 6 + 1 \cdot 5} = 0.143$$

Taking into consideration a very important fact that both the measures does not identify optimally modular structure identically, then, both the indicators need to be validated to turn one of them (if at all possible) into a true evaluation instrument. For this purpose, well known *Optimal modularity* index described in the next section is adapted to be applicable as standard reference method to identify optimally modular ALS from alternative ones.

### Adaptation of optimal modularity index to the ALS networks

The classical index of optimal modularity Q developed by Newman and Girvan^8^ quantifies modularity of the community structures in social and biological networks which are modelled as undirected graphs. This indicator considers important feature of modular systems that the great majority of interactions occur within modules and only a few interactions occur between modules. The algorithm was reinterpreted by several authors (see, e.g.^66–68^) in order to endorse its exploitation for different kinds of modular systems that can be decomposed into structurally independent parts. As ALSs are also decomposable into independent parts, it is a priori assumed that Q index should be able to identify optimal ALS from alternative options. The formula to quantify optimal modularity level of unweighted directed network (Qd) can be in simple form expressed as follows:

10 $$Qd = \mathop \sum \limits_{s = 1}^{n} \left( {\frac{ls}{L} - \frac{{ws^{out} \cdot ws^{in} }}{{L^{2} }}} \right),$$

where *n* is the number of modules, *L* is the number of all edges in the network, *ls* is the number of internal edges in module *s*,

*ws*_*out*_ is the number of output edges in module *s*, *ws*_*in*_ is the number of input edges in module *s*.

**Figure 4:**
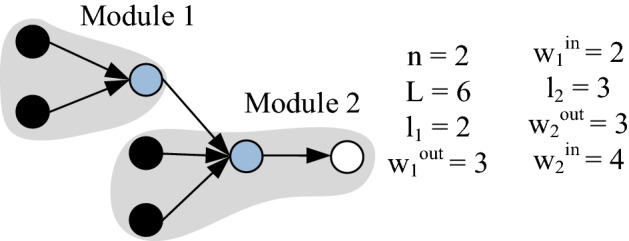
Parametrization of the selected graph from the viewpoint of modularity level quantification.

Principally, this indicator is applicable for any ALSs. For example, the modularity level of the process structure in Fig. 4 equals 0.33.

The higher values obtained by applying these three measures (Om_1_, Om_2_ and Qd) imply better partition of operations allocated into workstations.

### Testing and comparison of optimal modularity measures on assembly line models

In order to find out whether or not the proposed optimality modularity measures can be considered valid and reliable means of assessing ALS models, the two following computational tests were carried out:

1. To verify whether or not at least one of the two optimal modularity measures Om_1_ and Om_2_ identifies an optimal ALS within individual classes of ALSs uniformly or almost uniformly with Qd.
2. To verify whether or not fully symmetric ALSs are identified through Om_1_, Om_2_ and Qd as optimal or near optimal modular architectures.

At first, ALSs of the classes#4-8 will be explored as the test instances for the test No. 1.

At second, ALSs with the even number of input components will be used as the test graphs for the experiment No. 2.

The tested graphs of ALSs of class#4 are shown in Fig. 1, the graphs of ALSs of classes#5-7 are available in Appendix 1, while the graphs of ALSs of class#8 can be found in work of^69^.

For the testing, the formulas (8), (9) and (10) will be applied. The obtained values for the ALSs of the classes#4-7 are shown in Appendix 2, and the enumerated values for ALSs of the class#8 are available in Appendix 3, noting that graphs for all the ALS classes are positioned in ascending order according to Om_1_.

By analyzing the obtained values regarding the test No. 1, it can be stated that:

ALS class#4—the optimal ALS (structure No. 4) was uniformly identified through all the three optimality measures (Om_1_, Om_2_ and Qd).

ALS class#5—indicators Om_1_ and Qd identically determined structure No. 8 as optimal ALS from the alternative ones, while Om_2_ assigned structures No. 10 and No. 11 as the optimal ALSs.

ALS class#6 –structure No. 10 was determined to be optimal ALS through Om_1_ and Qd, while Om_2_ assigned as optimal structures the different two ALSs, i.e., structures No. 24 and No. 25.

ALS class#7—by using Om_1_ and Qd were obtained different 1st best optimal solutions, but according to both the indicators, network No. 4 presents, in the worst case, the second best solution from the 90 alternatives. However, according to Om_2_, the best optimal solution (network No. 31) differs from the 1^st^ and 2^nd^ best solutions by indicators Om_1_ and Qd.

ALS class#8—Indicators Om_1_ and Qd identically determined structure No. 257 as the optimal ALS, while Om_2_ assigned different 1^st^ best solution than Om_1_ and Qd.

When analyzing the obtained values regarding the test No. 2, one can state that:

ALS class#4—the fully symmetric structure No. 4 was uniformly identified as the optimal ALS by all the three optimal modularity indicators.

ALS class#6—through Om_1_ and Qd measures the fully symmetric structure No. 10 was uniformly identified as optimal modular ALS. Through Om_2_ measure, the two non-symmetric structures No. 24 and No. 25 were identified as optimal modular ALSs.

ALS class#8—the fully symmetric structure No. 257 was uniformly identified as the optimal ALS by Om_1_ and Qd, but according to Om_2_ the non-symmetric structures No. 58 and 214 were identified as optimal ones.

To summarize the obtained results from the testing, the following comparison is provided in Table 3:

**Table 3:** Summarized results from tests.

Optimality indicators	Test No. 1					Test No. 2		
	ALS structures					ALS structures		
	Class #4	Class #5	Class #6	Class #7	Class #8	Class #4	Class #6	Class #8
Om_1_	✓✓	✓✓	✓✓	✓	✓✓	✓✓	✓✓	✓✓
Om_2_	✓✓	–	–	–	–	✓✓	–	–
Qd	✓✓	✓✓	✓✓	✓	✓✓	✓✓	✓✓	✓✓

✓✓—fully satisfied results; ✓—partially satisfied results.

As can be seen from the comparison in Table 3, both the optimal modularity measures (Qd and Om_1_) uniformly identify the optimal ALSs within classes#4, 5, 6 and 8. Moreover, both the measures equally identified the fully symmetric ALSs within classes#4, 5, 6 and 8 as optimally modular ones. In contrary, indicator Om_2_ identified the same optimal ALS than Qd and Om_1_ indicators within class#4, but this class consists only from five alternative ALSs.

Based on the results, indicators Qd and Om_1_ would almost equally well serve the same purpose, while Om_2_ indicator seems to be rather not suitable for the given purpose, and therefore Om_2_ will not be further explored here.

Especially important finding is that symmetrical ALSs better reflect optimal modularity criteria than non-symmetrical ones.

This finding confirms the assertion of Efatmaneshnik and Ryan^7^ that optimal modularization of the structure can be achieved through symmetrical distribution of its modules. This led us to identify some useful properties and rules of the structural optimality measures described in the subsequent section.

## Findings and theoretical implications

Delving deeper into the mechanism of the two optimal modularity concepts expressed by formulas (8) and (10), one can analytically extract from them the following two common features:

(i) Optimal modularity of ALS is a function describing a relation between out-put/input edges and internal nodes of the given structure.
(ii) Both the two measures (Om_1_ and Qd) confirm earlier articulated positive effects of network symmetry on the optimality network measures. The effects of network symmetry are meant in the sense that graphs with a very low modularity seem to be substantially less symmetric^61^.

**Figure 5:**
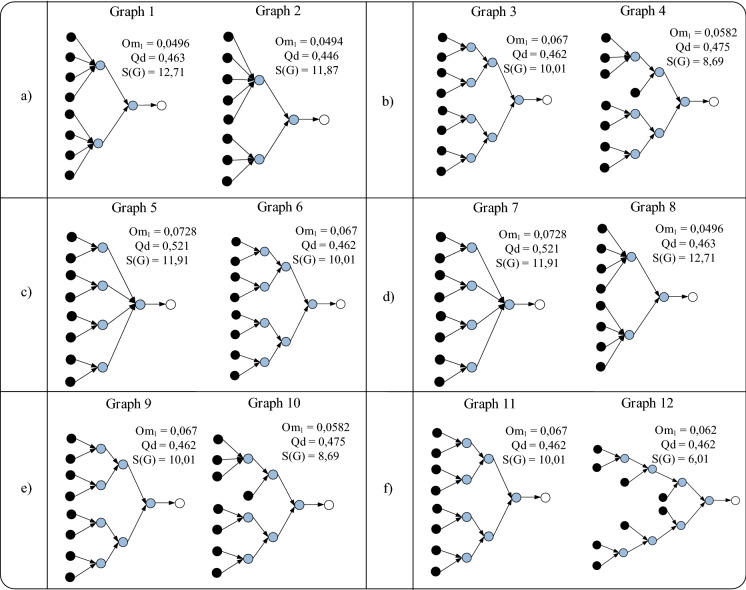
Comparison of the selected alternative ALSs by employing Om_1_ and S(G) measures.

In order to bring more insight and understanding about the relation between symmetry and optimal modularity, selected pairs of ALSs, depicted in Fig. 5, will be compared and analysed by using the three measures, i.e., Om_1_, Qd and S(G).

When comparing and analysing the obtained values depicted in Fig. 5, three main findings are worth noting here:

1. When comparing two or more assembly line structures of the same class with an even number of input components, then fully symmetric ALS better reflects the optimal modularity requirements than similarly bifurcated non symmetric ALS.

To demonstrate this finding, the following comparison of the two pairs of alternative ALSs is provided in Fig. 5a,b. As can be seen in these two subfigures, the both bifurcated non symmetric graphs, i.e., Graph 2 and Graph 4 are topologically similar to their symmetric pendants, while Graph 1 and Graph 3 are better modular than the non symmetric ones.

2. If we have two or more alternative assembly line structures whose models represent bifurcated fully symmetric graphs, then a graph with a higher number of internal nodes at the second level of the hierarchy better reflects the optimal modularity requirements than that or those with a lower number.

Illustrative examples of the comparison of the two pairs of alternative ALSs is provided in Fig. 5c,d, where Graph 6 and Graph 8 consist of the two internal nodes at the second level, while their pendants (Graph 5 and Graph 7) consist of the four internal nodes at the same hierarchy level. Based on the obtained Om_1_ values, it is clear that Graph 5 is better modular than Graph 6, and Graph 7 is better modular than Graph 8. Moreover, as expected, Graph 5 is better symmetric than Graph 6, but on the contrary, Graph 7 is less symmetric than Graph 8. The symmetry comparison of the pairs of Graphs from Fig. 5a–d shows that the symmetry indicator S(G) is helpful in determining optimal modularity network, but it should not substitute optimal network modularity measures due to its complementary role in combination with the targeted indicators.

3. If we have two or more alternative assembly line structures with the same number of internal nodes at the second level, then a structure with a higher number of internal nodes at the third level better reflects the optimal modularity requirements than an ALS structure with a comparable lower number.

A comparison of the two pairs of alternative ALSs is provided in Fig. 5e,f to illustrate that Graph 9 better meets the optimal modularity requirements than Graph 10, and Graph 11 better satisfies the expectations for optimal modularity than Graph 12. Moreover, the obtained values of the Symmetry index support the results of the comparison of the two pairs of ALSs.

When analyzing all the obtained values from this comparison, it can be stated that proposed generalizations confirm previous insights^62^ that symmetry induces equivalence classes of partitions. In other words, these findings also suggest that symmetrical ALSs tend to be optimally modular.

## Practical applicability and limitations

In this section we apply the two different process optimality measures, namely RNM—i.e. relative modularity indicator, and Om_1_—the proposed optimal modularity indicator to real assembly process case. Our intent here is two-fold. First, we want to show that real assembly processes more or less follow graphical patterns of multifurcuting rooted directed convergent trees which were used in this work for specifying the formal models of ALSs. Our second purpose is to demonstrate on the selected practical example that maximum relative modularity doesn’t equal the optimal positive state of the process structure from a modularity viewpoint. A bulldozer case study from the works^45,70,71^ is presented here to demonstrate the above mentioned intentions. Based on decomposition of the assembly system, there are 14 major components of the product that are assembled in several different ways. At first we selected eight of them (see Appendix 4a) which are presented in the mentioned previous research works. When analysing their structures, it is clear that they follow the theoretical graphical patterns of multifurcuting rooted directed convergent trees. At a high level, the product consists of the following components, which are frame assembly (1), case (2), brake (3), drive (4), plant carrier (5), transmission (6), roll-over (7), fender (8), platform (9), engine (10), fan (11), pin assembly (12), bogie assembly (13), and track-roller frame (14).

Then, relative modularity values and optimal modularity values were calculated using the both above mentioned indicators (RNM, Om_1_) as shown in table in Appendix 4b. From this table one can see that the alternative No. 8 presents the process structure with maximum relative modularity, while the alternative No. 7 is identified as optimally suited to satisfy modularity requirement according to indicator Om_1_. When subsequently comparing the two ALS alternatives No. 7 and No. 8 in relation to symmetry properties by using formula (7), coincidentally, ALS No. 7 has higher symmetry than ALS No. 8.

Regarding limitations of the proposed methodology, we remark that optimal ALS in real conditions can be identified only from assumed alternatives which do not include all of the possible alternatives respecting precedence constraints and other technical or technological conditions. For example, in above mentioned study were considered eight realistic alternatives, but probably it would be possible to identify greater number of realistic alternatives. In such case, optimal modular ALS identified from eight alternatives, could be different than optimal modular ALS identified from all the possible alternatives.

## Conclusions

Coming back to the research questions set out in the introduction section, it is possible to formulate the following answers.

Answer to RQ 1: Based on the results of the all comparisons of the concurrent indicators Qd and Om_1_ (together 401 computational experiments for each of the indicators) it can be stated that for the classes of ALSs with even number of input components (class #4, #6, #8, etc.) both indicators provide the same results. But, for the higher classes of ALSs with odd number of input components (starting from class #7) Qd method identifies more than one ALS with optimal modularity. In other words, Qd indicator seems to be less sensitive in case of the higher classes of ALSs with odd number of input components than Om_1_ indicator.

Answer to RQ 2: As the results of all the comparisons of the concurrent indicators Qd and Om_1_ showed, indicator Om_1_ can be effectively applied to identify optimal process structure from the available set of ALS alternatives. Moreover, this indicator can be directly used to quantify modularity level from a graphical representation of ALS, while Qd method needs an additional graphic adaptation of ALS as shown in “Adaptation of optimal modularity index to the ALS networks” Section.

The research reported in this study has yielded also some useful findings, conclusions and implications for particular design problem including the following.

First, it gives a hint on how the structural modularity of assembly process affects its structural complexity, and how important is to select the adequate quantitative measures for investigation of this relationship.

Second, the problem of optimal configuration of ALSs with respect to modularity can only be effectively explored with simultaneous measurements of relative modularity.

Third, the adapted *optimal modularity* index Qd can be reliably used to identify optimal ALS from among a pre-defined set of alternatives for an even number of input components, and also for ALSs of class#5.

Fourth, the proposed optimal modularity measure (Om_1_) can be effectively used to identify optimal ALS from among a predefined set of alternatives without any significant limitations. Other important advantage of this indicator is that provides a very sensitive detection of differences in the modularity between alternative ALSs.

Fifth, it provides evidence that more symmetric ALSs of the same class tend to be more optimally modular, than less symmetric ones.

Taking into account increasing requirements on assembly process modularity, then possible practical implications of the proposed method can be seen in the following:

- The construct of this method offers more insight into (more or less) causal relation between optimal modularity of process structures and module level complexity. It could help designers to indirectly manage process structure complexity through the proposed indicator in order to obtain a suitable level of complexity of each workstation to avoid working conditions which is neither causing physical or mental stress nor demotivating human assembly operators.
- Assuming that optimal process structure settings can lead to consistent end-product quality and higher process efficiency, then such possible effects are in line with sustainable goals of manufacturing systems and increase their resilience to market disturbances.

There is no doubt that the problem of detecting optimal structural modularity is one of the most challenging issues in the study of network systems. Therefore, further research is expected regarding implications of modular design choices on process performance characteristics. Moreover, structural process modularity has other important implications, e.g., for organizational coordination, process control and so forth. Finally, further effort will be needed in implementation of the proposed method in a real-world environment.

## Supplementary Information

Supplementary Information 1.

Supplementary Information 2.

Supplementary Information 3.

Supplementary Information 4.

## Data Availability

All data generated or analysed during this study are included in this published article.

## References

[1] Hu SJ, Zhu X, Wang H, Koren Y (2008). Product variety and manufacturing complexity in assembly systems and supply chains. CIRP Ann..

[2] Shamsuzzoha AHM, Helo PT (2011). Information dependencies within product architecture: Prospects of complexity reduction. J. Manuf. Technol. Manag..

[3] Ethiraj SK, Levinthal D (2004). Modularity and innovation in complex systems. Manage. Sci..

[4] Hilger K, Ekman M, Fiebach CJ, Basten U (2017). Intelligence is associated with the modular structure of intrinsic brain networks. Sci. Rep..

[5] Fredriksson P (2006). Operations and logistics issues in modular assembly processes: Cases from the automotive sector. J. Manuf. Technol. Manag..

[6] Bonjour E, Deniaud S, Dulmet M, Harmel G (2009). A fuzzy method for propagating functional architecture constraints to physical architecture. J. Mech. Design. Am. Soc. Mech. Eng..

[7] Efatmaneshnik M, Ryan MJ (2016). On optimal modularity for system construction. Complexity.

[8] Newman MEJ, Girvan M (2004). Finding and evaluating community structure in networks. Phys. Rev. E.

[9] Guimera R, Nunes Amaral LA (2005). Functional cartography of complex metabolic networks. Nature.

[10] Chiriac N, Hölttä-Otto K, Lysy D, Suk Suh E (2011). Level of modularity and different levels of system granularity. J. Mech. Design.

[11] Metzler T, Mosch M, Lindemann U (2013). Meta-design catalogs for cognitive products. ICoRD’13 Lect. Not. Mech. Eng..

[12] Parraguez, P. Process Modularity Over Time: Modeling Process Execution as an Evolving Activity Network.

[13] Blecker T, Abdelkafi N (2006). Complexity and variety in mass customization systems: Analysis and recommendations. Manag. Decis..

[14] Newman MEJ (2006). Modularity and community structure in networks. Proc. Natl. Acad. Sci..

[15] Duch J, Arenas A (2005). Community detection in complex networks using extremal optimization. Phys. Rev. E.

[16] Nematzadeh A, Ferrara E, Flammini A, Ahn YY (2014). Optimal network modularity for information diffusion. Phys. Rev. Lett..

[17] Kashkoush M, ElMaraghy H (2017). Designing modular product architecture for optimal overall product modularity. J. Eng. Des..

[18] Sakundarini N, Taha Z, Ghazilla RAR, Abdul-Rashid SH (2015). A methodology for optimizing modular design considering product end of life strategies. Int. J. Precis. Eng. Manuf..

[19] Abdelkafi, N. Variety induced complexity in mass customization: Concepts and management. (2008).

[20] Brusoni S, Marengo L, Prencipe A, Valente M (2007). The value and costs of modularity: A problem-solving perspective. Eur. Manag. Rev..

[21] Ulrich K (1995). The role of product architecture in the manufacturing firm. Res. Policy.

[22] Gualandris J, Kalchschmidt M (2013). Product and process modularity: Improving flexibility and reducing supplier failure risk. Int. J. Prod. Res..

[23] Dolgui A, Ivanov D, Sokolov B (2020). Reconfigurable supply chain: The X-network. Int. J. Prod. Res..

[24] Brahimi N, Dolgui A, Gurevsky E, Yelles-Chaouche AR (2019). A literature review of optimization problems for reconfigurable manufacturing systems. IFAC-PapersOnLine.

[25] Kusiak A (2010). Integrated product and process design: A modularity perspective. J. Eng. Des..

[26] Yelles-Chaouche AR, Gurevsky E, Brahimi N, Dolgui A (2021). Reconfigurable manufacturing systems from an optimisation perspective: A focused review of literature. Int. J. Prod. Res..

[27] Parker, D. B. Modularity and complexity: An examination of the effects of product structure on the intricacy of production systems. Unpublished doctoral dissertation, Michigan State University, East Lansing, MI. (2010).

[28] Suh NP, Cochran DS, Lima PC (1998). Manufacturing system design. CIRP Ann..

[29] Vickery SK, Koufteros X, Dröge C, Calantone R (2015). Product modularity, process modularity, and new product introduction performance: Does complexity matter?. Prod. Oper. Manag..

[30] Fixson SK (2007). Modularity and commonality research: Past developments and future opportunities. Concurr. Eng..

[31] Piran FAS, Lacerda DP, Camargo LFR, Viero CF, Teixeira R, Dresch A (2017). Product modularity and its effects on the production process: An analysis in a bus manufacturer. Int. J. Adv. Manuf. Technol..

[32] Xiao Y, Zhang H (2021). New product advantage infused by modularity: Do resources make a difference?. J. Prod. Innov. Manag..

[33] Jayaram J, Vickery S (2018). The role of modularity in the supply chain context: Current trends and future research directions. Int. J. Prod. Res..

[34] Zelditch ML, Goswami A (2021). What does modularity mean?. Evol. Dev..

[35] Frenken K, Mendritzki S (2012). Optimal modularity: A demonstration of the evolutionary advantage of modular architectures. J. Evol. Econ..

[36] Blackenfelt, M. Managing complexity by product modularisation, Doctoral dissertation, Royal Institute of Technology, Sweden (2001).

[37] AlGeddawy T, ElMaraghy H (2013). Optimum granularity level of modular product design architecture. CIRP Ann..

[38] AlGeddawy T, Samy SN, ElMaraghy H (2017). Best design granularity to balance assembly complexity and product modularity. J. Eng. Des..

[39] Yao YY (2003). Probabilistic approaches to rough sets. Expert. Syst..

[40] Modrak V, Soltysova Z (2021). Development of the modularity measure for assembly process structures. Math. Probl. Eng..

[41] Modrak V, Soltysova Z (2018). Process modularity of mass customized manufacturing systems: Principles, measures and assessment. Procedia CIRP.

[42] Kashtan N, Alon U (2005). Spontaneous evolution of modularity and network motifs. Proc. Natl. Acad. Sci. USA.

[43] Kirschner M, Gerhart J (1998). Evolvability. Proc. Natl. Acad. Sci. USA.

[44] Wagner GP (2005). Robustness and Evolvability in Living Systems, Princeton.

[45] Alkan B, Bullock S, Galvin K (2021). Identifying optimal granularity level of modular assembly supply chains based on complexity-modularity trade-off. IEEE Access.

[46] Shoval S, Efatmaneshnik M, Ryan MJ (2017). Probabilistic approach to modular assembly. IFAC-PapersOnLine.

[47] Mehrsai A, Karimi HR, Thoben KD (2013). Integration of supply networks for customization with modularity in cloud and make-to-upgrade strategy. Open Access J. Syst. Sci. Control Eng..

[48] Sharmelly R, Ray P (2015). Organizational capabilities for mass market innovations in the emerging economies: Insights from an automobile firm in India. J. Comp. Int. Manag..

[49] Park J, Park J, Kim J (2000). A generic event control framework for modular flexible manufacturing systems. Comput. Ind. Eng..

[50] On-line Encyclopedia of Integer Sequences (OEIS), https://oeis.org/A000669 (2022).

[51] Coronado TM, Francesc Rosselló AM, Rotger L (2020). On Sackin’s original proposal: The variance of the leaves’ depths as a phylogenetic balance index. BMC Bioinform..

[52] Tran TD, Kwon YK (2013). The relationship between modularity and robustness in signalling networks. J. R. Soc. Interface..

[53] Larses, O. & Blackenfelt, M. Relational reasoning supported by quantitative methods for product modularization. In *DS 31: Proceedings of ICED 03, the 14th International Conference on Engineering Design Stockholm* 347–348 (2003).

[54] Min G, Suh ES, Hölttä-Otto K (2016). System architecture, level of decomposition, and structural complexity: Analysis and observations. J. Mech. Des..

[55] Goeman JJ, Solari A (2010). The sequential rejection principle of familywise error control. Ann. Stat..

[56] Guenov, M. D. Complexity and cost effectiveness measures for systems design. In *Manufacturing Complexity Network Conference* 1–13, (2002).

[57] Sinha K, Weck OLD (2013). A network-based structural complexity metric for engineered complex systems. Proc. IEEE Int. Syst. Conf. (SysCon).

[58] Crippa, R., Bertacci, N. & Larghi, L. Representing and Measuring Flow Complexity in the Extended Enterprise: The D4G Approach. RIRL International Congress for Research in Logistics (2006).

[59] Kubota FI, Gontijo LA, Miguel PAC (2012). Design modularity: Identification of benefits and difficulties through a bibliographical analysis in the perspective of automotive assemblers and suppliers. Prod. Manag. Dev..

[60] Yogev, O., Shapiro, A. A. & Antonsson, E. K. Modularity and symmetry in computational embryogeny. In *Proceedings of the 10th annual conference on Genetic and evolutionary computation* 1151–1152, 10.1145/1389095.1389323 (2008).

[61] Ball F, Geyer-Schulz A (2018). How symmetric are real-world graphs? A large-scale study. Symmetry.

[62] Ball F, Geyer-Schulz A (2018). Invariant graph partition comparison measures. Symmetry.

[63] Mowshowitz A, Dehmer M (2010). A symmetry index for graphs. Symmetry Cult. Sci..

[64] Sinha K, Suh ES (2018). Pareto-optimization of complex system architecture for structural complexity and modularity. Res. Eng. Design.

[65] Hinchey M, Coyle L (2012). Conquering Complexity.

[66] Alon U (2020). An Introduction to Systems Biology: Design Principles of Biological Circuits.

[67] Arenas A, Duch J, Fernández A, Gómez S (2007). Size reduction of complex networks preserving modularity. New J. Phys..

[68] Barabási AL (2013). Network science. Philos. Trans. R. Soc. A: Math. Phys. Eng. Sci..

[69] Modrak V (2017). Mass Customized Manufacturing: Theoretical Concepts and Practical Approaches.

[70] Nepal B, Monplaisir L, Famuyiwa O (2012). Matching product architecture with supply chain design. Eur. J. Oper. Res..

[71] Graves SC, Willems SP, de Kok AG, Graves SC (2003). Supply chain design: Safety stock placement and supply chain configuration. Handbooks in Operations Research and Management Science, Supply Chain Management: Design.

