# Supplementary material for: Exploration of the optimal modularity in assembly line design
Source: Sci Rep. 2022 Nov 27;12:20414. doi: 10.1038/s41598-022-24972-2 (PMC9701789; doi:10.1038/s41598-022-24972-2)
Supplement: Supplementary file 1 — Supplementary Information 1. [file 41598_2022_24972_MOESM1_ESM.docx]

**Appendix 1.** The graphs of the all possible alternative ALSs: a) of the class#5, b) of the class#6, c) of the class#7.
