# Supplementary material for: Exploration of the optimal modularity in assembly line design
Source: Sci Rep. 2022 Nov 27;12:20414. doi: 10.1038/s41598-022-24972-2 (PMC9701789; doi:10.1038/s41598-022-24972-2)
Supplement: Supplementary file 2 — Supplementary Information 2. [file 41598_2022_24972_MOESM2_ESM.docx]

**Appendix 2.** Comparison of Qd, Om_1_ and Om_2_ indicators through their application to ALSs of a) class#4, b) class#5, c) class#6; d) class#7.
