# Supplementary material for: Exploration of the optimal modularity in assembly line design
Source: Sci Rep. 2022 Nov 27;12:20414. doi: 10.1038/s41598-022-24972-2 (PMC9701789; doi:10.1038/s41598-022-24972-2)
Supplement: Supplementary file 3 — Supplementary Information 3. [file 41598_2022_24972_MOESM3_ESM.docx]

**Appendix 3.** Comparison of the two optimal modularity measures through their application to ALSs of class#8.
