# Supplementary material for: Exploration of the optimal modularity in assembly line design
Source: Sci Rep. 2022 Nov 27;12:20414. doi: 10.1038/s41598-022-24972-2 (PMC9701789; doi:10.1038/s41598-022-24972-2)
Supplement: Supplementary file 4 — Supplementary Information 4. [file 41598_2022_24972_MOESM4_ESM.docx]

**Appendix 4.** Practical case study a) selected possible eight alternative ALSs; b) Obtained relative and optimal modularity values.
